# Chronic Microplastic Exposure and Cadmium Accumulation in Blue Crabs

**DOI:** 10.3390/ijerph19095631

**Published:** 2022-05-05

**Authors:** María Hernández-López, Diego Romero

**Affiliations:** Área de Toxicología, Facultad de Veterinaria, Campus de Espinardo, Universidad de Murcia, 30100 Murcia, Spain; m.hernandezlopez1@um.es

**Keywords:** accumulation, blue crab, cadmium, gill, haemolymph, hepatopancreas, microplastic, muscle

## Abstract

Aquatic ecosystems are severely threatened by the presence of a multitude of pollutants. In seas and oceans, the amount of plastics continues to increase and there is great concern about toxic element accumulation. Specifically, cadmium (Cd), a toxic metal, is highly relevant to public health safety due to its ability to accumulate in the internal tissues of crustaceans; likewise, microplastics (MPs) are emerging as pollutants capable of causing alterations in marine organisms. The aim of this study was thus to evaluate the accumulation and distribution of Cd in the tissue of blue crabs (*Callinectes sapidus*) chronically exposed to MPs (25 μg L^−1^). In total, 24 crabs were exposed in water for 118 days to 2 types of MPs (virgin and oxidised). During the final 21 days of the experiment, the crabs were fed with tuna liver, a viscera in which Cd accumulates (mean of 7.262 µg g^−1^). The presence of MPs caused no changes in Cd concentrations in either the haemolymph or tissues (hepatopancreas, gills, and muscles) of the crabs, although for oxidised MPs, there was a positive correlation between Cd concentrations in the hepatopancreas and muscles, a relevant finding for food safety.

## 1. Introduction

Marine contamination is a worldwide problem, and the presence of many types of pollutants in the sea has generated great social and scientific concern as many products of marine origin are consumed on a regular basis throughout the world. For years, urban and industrial development (anthropogenic activities) has led to the presence —and in recent years, an increase—in toxic elements in marine ecosystems, which includes the growing amounts of the so-called ‘emerging pollutants’ that include microplastics (MPs).

Cadmium (Cd) is a highly toxic trace element, ubiquitous in the environment but present in only low concentrations in the Earth’s crust. It occurs naturally as the result of the decomposition of rocks, forest fires, and even volcanic eruptions [1,2,3,4,5]. However, the main origin of environmental contamination by Cd is anthropogenic since it is generated as a by-product when smelting other metals including zinc (Zn), to which it is closely related, copper (Cu), and lead (Pb) [5,6]. It has also been used for years in industry as an anticorrosive, in the manufacture of alkaline batteries, in pigments and paints, and for welding [7]. It has no biological function in living organisms but is found in all ecosystems, both aquatic and terrestrial, and can be displaced by air and rain [8]. In this way, Cd enters the food chain of living organisms and can provoke a wide range of harmful effects. In vertebrates, it accumulates in organs such as the kidney and liver but is also found in invertebrates, such as plants and algae [9,10]. Given that feeding on Cd-contaminated food can cause serious health issues, over the years, many reports, legal regulations, and recommendations have been published on this subject by health authorities worldwide [11,12,13,14,15,16].

Another problem for ecosystems is the massive use of plastics, which are the main source of marine litter, and which are mainly generated by anthropogenic activities [17]. According to some estimates [18], the number of plastic particles or microplastics (MPs) originating from various sources found in the oceans may be as many as 51 trillion. Primary MPs enter the marine environment directly, for example, as manufactured cosmetic products (e.g., facial cleansers or toothpastes) or as industrial waste and synthetic textiles. According to some authors [19,20,21], wastewater from synthetic laundry can contain over 100 fibres per litre of water. Secondary MPs are the most abundant plastics and represent 69–81% of those present in marine ecosystems. They are the product of the degradation and fragmentation of large plastic objects such as fishing nets, bottles, and plastic bags [22]. Irrespective of their origin, MPs damage marine environments as they are ingested and accumulated by all types of living organisms and therefore enter the food chain [23].

The sentinel organisms most commonly used in marine ecotoxicological studies are algae, benthic invertebrates (bivalves, annelids, crustaceans, etc.), and pelagic species, usually fish [24]. The blue crab (*Callinectes sapidus*) is of great interest in toxicological research due to its characteristics, habits, and ease of handling, and has been used in several experimental studies [25,26,27]. This crustacean (decapod) is regarded as an invasive species along Mediterranean coasts as it originates from the Atlantic Ocean. As an opportunistic scavenger, it preys on organisms on the bottom of seas and rivers and consumes the carrion of various species, thereby ingesting the pollutants they contain.

Given the importance of these two types of pollutants (Cd and MPs), the main objective of this study was to explore the impact that MPs have on Cd tissue concentrations and distribution in blue crabs, a species that is increasingly being consumed by humans in the Mediterranean Basin.

## 2. Material and Methods

In accordance with European legislation (Directive 2010/63/UE), the procedures employed did not require ethical permissions.

### 2.1. Capture and Acclimatisation

**Figure 1:**
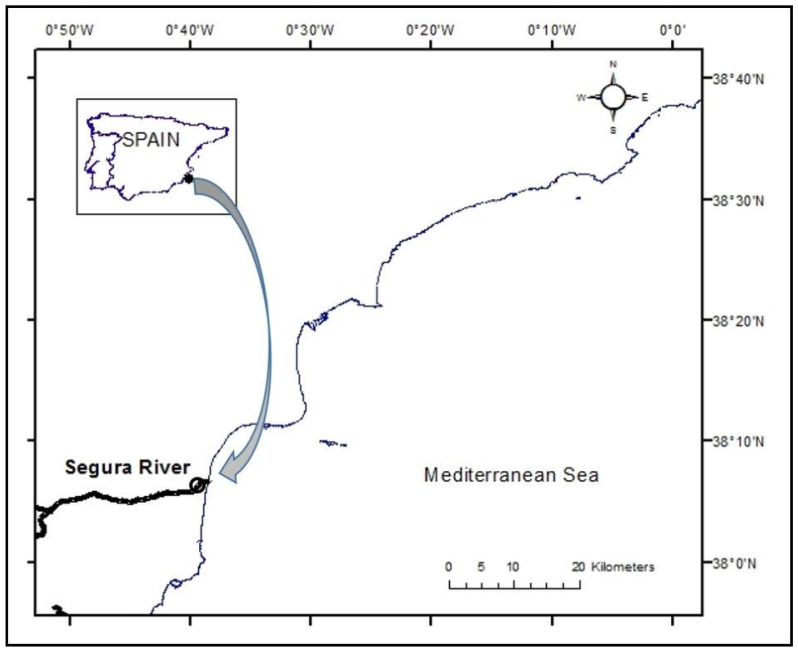
Location of sampling area (Azud de San Antonio, Guardamar del Segura, Spain).

**Figure 2:**
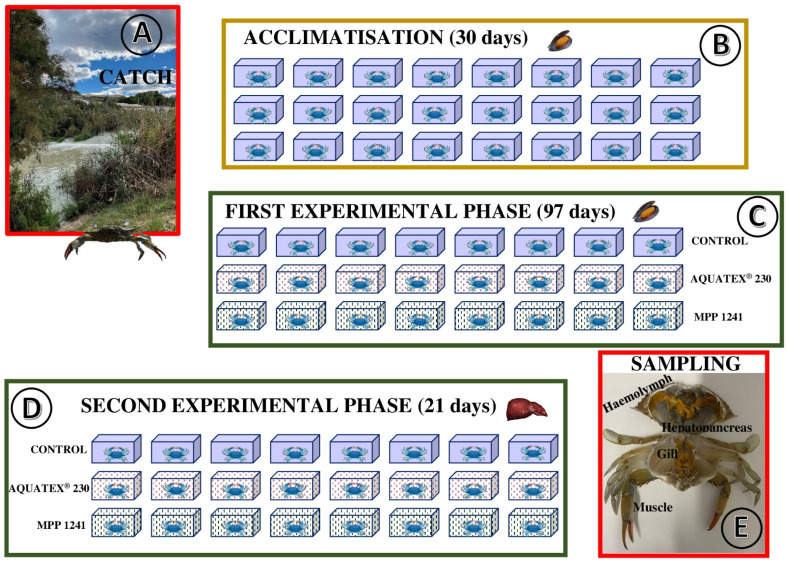
Experimental procedure. (**A**): catch (Azud of San Antonio, Guardamar del Segura, Spain); (**B**): acclimatisation (no MPs and crabs fed on mussels); (**C**): first experimental phase (MPs and crabs fed on mussels); (**D**): second (MPs and crabs fed on tuna liver) experimental phase; (**E**): sampling.

The crabs (*n* = 32) were captured in October 2020 at Azud de San Antonio (UTM, 705558X-4220269Y; Guardamar del Segura, Alicante province, Figure 1) at the mouth of the river Segura in fyke nets baited with sardines and mussels (Figure 2A). The nets were placed on the shore and left for 3–4 h; captured crabs were individually placed in water from the area for transport to the laboratory. The crabs were classified into four homogeneous groups according to the following biometric data: weight, body length (distance between the abdominal segment and rostrum), and width (distance between lateral spines). Of the 32 crabs, 8 (‘zero-hour’) were euthanised after the collection of their faeces by hypothermia (−20 °C for 10–15 min). Prior to dissection for sampling, a haemolymph sample (0.5–1.0 mL) was extracted from these specimens using an insulin syringe inserted directly into the heart through the joint between the carapace and the fifth limb. Then, the carapace was opened with a scalpel and scissors, and samples of the hepatopancreas, muscle, and gills were collected, transferred to 1.50 mL microtubes, and stored at −20 °C until processing and chemical analysis. The faeces were washed 5 times in osmosis water, dried in an oven at 60 °C, and stored at room temperature until chemical analysis.

The remaining 24 specimens were acclimatised for 30 days (Figure 2B) in individual 6 L water tanks with the following characteristics: salinity 2 g L^−1^, osmolarity 55.0 ± 5.1, pH 7.2 ± 0.1, temperature 21.0 ± 0.5 °C, and continuous aeration and natural photoperiod (12 h of light and 12 h of darkness), with a change of the water every 3–4 days. During this period, the crabs were fed ad libitum (three times a week) with wild mussels (*Mytilus galloprovincialis*) intended for human consumption; the amount of food ingested was recorded for each specimen and intake.

### 2.2. Experimental Procedure

Once acclimatised, the experiment was carried out (Figure 2C,D) on three groups of eight crabs each: (1) control group, (2) group exposed to oxidised polyethylene (AQUATEX^®^ 230, Micro Powders INC, USA; mean particle size 36–40 μm, with a maximum of 63 µm), and (3) group exposed to virgin high-density polyethylene (MPP 1241, Micro Powders INC, Tarrytown, New York, USA; mean particle size 20–25 μm, with a maximum of 110 µm). In both cases, the MP concentrations were 25 μg L^−1^, which is the level of MPs predicted to be present in oceans in 2025 [28,29].

In the first phase of the experiment (97 days, Figure 2C), the crabs were fed ad libitum (three times a week) with wild mussels with no changes in the conditions described for the acclimatisation period (salinity, osmolarity, pH, temperature, aeration, and photoperiod). In the second phase (21 days, Figure 2D), the crabs were fed ad libitum (three times a week) with bluefin tuna (*Thunnus thynnus*) liver slaughtered for human consumption. The viscera of the adults of this fish can accumulate Cd concentrations of up to several parts per million [30,31]. In this phase, the environmental conditions indicated in the previous phases remained unaltered, although the water was changed every 48 h. During this period, faecal samples were collected, specifically, at the beginning, middle, and end of the phase. Due to their lightness, faeces from each group were pooled and processed as indicated for the zero-hour group. Finally, all 24 crabs were euthanised by hypothermia and samples were collected following the procedures described for the zero-hour crabs (Figure 2E).

### 2.3. Cd Analysis

Cadmium concentrations were determined using inductively coupled plasma optical emission spectrometry (ICP-OES, ICAP 6500 Duo, Thermo Scientific, Waltham, Massachusetts, USA). Haemolymph, hepatopancreas, gill, muscle, faeces, mussel, and liver samples were treated with 4 mL of trace mineral grade HNO_3_ and 1 mL H_2_O_2_ (69 and 33%, respectively, Suprapure, Merck, Darmstadt, Germany) in special Teflon tubes, heated with a microwave digestion system (UltraClave-Microwave Milestone^®^, Sorisole, Italy) at 220 °C for 20 min, and diluted to 10 mL with double distilled water. The limit of detection for the Cd was 0.001 µg g^−1^. For each sample, two readings were collected and the mean was used as the concentration value. To check for possible external metal contamination, one blank sample for every eleven samples was also analysed. The standard calibration (SCP Science, Montreal, Canada) was prepared in 4% nitric acid, taking UNE-EN ISO 11885 as a reference for the determination of elements by ICP-OES. The wavelength used was 214.438 nm; the uncertainty and recovery percentages for reference materials were 4.56 and 95.32, respectively. For haemolymph and tissues, Cd concentrations are expressed in micrograms per gram of wet weight (μg g^−1^, ww), while for faeces, they are expressed as dry weight (dw).

Cadmium concentrations were determined using inductively coupled plasma optical emission spectrometry (ICP-OES, ICAP 6500 Duo, Thermo Scientific, Waltham, Massachusetts, USA). Haemolymph, hepatopancreas, gill, muscle, faeces, mussel, and liver samples were treated with 4 mL of trace mineral grade HNO_3_ and 1 mL H_2_O_2_ (69 and 33%, respectively, Suprapure, Merck, Darmstadt, Germany) in special Teflon tubes, heated with a microwave digestion system (UltraClave-Microwave Milestone^®^, Sorisole, Italy) at 220 °C for 20 min, and diluted to 10 mL with double distilled water. The limit of detection for the Cd was 0.001 µg g^−1^. For each sample, two readings were collected and the mean was used as the concentration value. To check for possible external metal contamination, one blank sample for every eleven samples was also analysed. The standard calibration (SCP Science, Montreal, Canada) was prepared in 4% nitric acid, taking UNE-EN ISO 11885 as a reference for the determination of elements by ICP-OES. The wavelength used was 214.438 nm; the uncertainty and recovery percentages for reference materials were 4.56 and 95.32, respectively. For haemolymph and tissues, Cd concentrations are expressed in micrograms per gram of wet weight (μg g^−1^, ww), while for faeces, they are expressed as dry weight (dw).

### 2.4. Data Analysis

The descriptive statistics are shown as mean ± standard deviation; the minimum and maximum values for Cd concentrations are also given. For data below the detection limit, the international guidelines established for this purpose were followed (the mean limit approach, whereby a value below the limit of detection is considered to be equal to half its value; [32]). Mussel and liver consumption was calculated for each specimen based on the amount of food ingested (food offered minus food rejected) to calculate the percentage of food in relation to body weight.

To test for the normality of the data, the Shapiro–Wilk test was used. Homogeneity of variances was checked using Levene’s test and the parametric ANOVA, with Tukey’s and Games Howell tests used as post-hoc tests. The biometric measurements of the crabs (at the beginning of the experiment) from the different groups (zero-hour and the groups that consumed tuna liver) were compared. As well, comparisons were made of (1) the percentage of mussel and liver ingested; (2) the concentrations of Cd in haemolymph, hepatopancreas, gills, and muscles; and (3) the concentrations of Cd in faeces. Finally, within each group in the experiment, haemolymph and tissue Cd concentrations were also compared.

The correlation study was carried out using Pearson’s test. In the zero-hour group, the correlation between biometric measurements of the crabs and Cd concentrations in the hepatopancreas was analysed. For each group from the experiment, the correlation between the biometric measurements and the concentrations of Cd in the haemolymph and tissues was also evaluated, as well as the correlation between the concentrations of Cd (in haemolymph and tissues) and the percentage of food (tuna liver). Finally, also for each of the groups in the experiment, the correlation between haemolymph, hepatopancreas, gills, and muscles (Cd concentrations) was analysed.

In all tests, a *p*-value of less than 0.05 was considered statistically significant. Statistical analysis was performed with IBM SPSS Statistics 24.0.

## 3. Results

The biometric measurements of the crabs are shown in Table 1. Statistical comparisons of the groups revealed that there were no significant differences for any of the variables (weight, length, and width).

The Cd concentration in the mussels (acclimatisation and first phase of the experiment) was 0.119 ± 0.006 µg g^−1^, while the concentration of Cd in the tuna liver was 7.262 ± 2.503 µg g^−1^.

In the experiment, the crabs from the control group consumed (relative to their body weight) 11.78 ± 2.75% (mussel) and 4.53 ± 2.35% (liver); the crabs exposed to AQUATEX^®^ 230 consumed 12.28 ± 1.65% (mussel) and 5.61 ± 1.40% (liver); and those exposed to MPP 1241 consumed 11.36 ± 3.13% (mussel) and 4.72 ± 1.68% (liver). No statistically significant differences between these groups were observed.

In zero-hour crabs, although the hepatopancreas samples had Cd above the detection limit, no Cd was detected in any haemolymph, gill, or muscle samples. In crabs fed on tuna liver (experiment groups), Cd concentrations were above the detection limit; in addition, the percentage of haemolymph samples above this limit in this group was 95.80% (all samples except one from the control group).

The Table 2 shows the Cd concentrations in haemolymph and tissues. In the control group and crabs exposed to MPs, the hepatopancreas had the highest Cd concentrations, while the lowest concentrations were found in the muscles and haemolymph. The concentrations of Cd in the hepatopancreas showed statistically significant differences between zero-hour crabs and those fed with tuna liver (experimental groups). No statistically significant differences in Cd concentrations (for any tissue) between groups fed with tuna liver were found. Statistical differences in Cd concentrations between tissues (for the same group) are indicated with capital letters in superscript.

Cadmium was only detected in faeces of crab that had consumed tuna liver. Although the highest Cd concentrations were found in the MPP-exposed group, no statistically significant differences between the experimental groups were detected.

Cadmium concentrations in the hepatopancreas were positively correlated with the percentage of liver intake in the three exposure groups: r = 0.751 and *p* < 0.05 (control), r = 0.717 and *p* < 0.05 (AQUATEX^®^ 230), and r = 0.952, *p* < 0.01 (MPP 1241). A correlation of Cd concentrations between hepatopancreas and gills (control group, r = 0.782, *p* < 0.05), between hepatopancreas and muscles (AQUATEX^®^ 230 and MPP, r = 0.733 and r = −0.717 respectively; *p* < 0.05 in both cases), and between haemolymph and gills (AQUATEX^®^ 230, r = 0.853, *p* < 0.05) were detected. Finally, no relationship between the biometric measurements and Cd concentrations in the haemolymph or tissues was observed.

## 4. Discussion

Cadmium concentrations in the zero-hour crabs were slightly higher (hepatopancreas) or slightly lower (gills and muscles) than those found in previous studies of the same species from the same ecosystem [33], and lower than those reported from other areas [34,35,36,37,38,39,40,41]. In the zero-hour group, the hepatopancreas had the highest Cd concentrations, which agrees with findings reported by other authors [34,35,36]. For haemolymph, no previous studies of *C. sapidus* could be found to act as a comparison, although according to Ortega et al. [42], concentrations of Cd in this matrix in *C. danae* were similarly low.

Regarding food, Cd concentrations in mussels (acclimatisation and in the first phase of the experiment) were similar to those reported by Falcó et al. [43] and Zhelyazkov et al. [44] in mussels for human consumption. In the case of tuna liver, Cd concentrations were similar or lower than those found in wild tuna [45,46]. Therefore, the food we supplied to crabs in the bioassay could be considered to be natural and likely to be present in many ecosystems.

### 4.1. Effects of Microplastics

Several authors have shown how MPs affect marine organisms such as fish, amphipod crustaceans, mussels, and marine worms [47]. In most cases, a decrease in food consumption due to the presence of these contaminants—which does not coincide with our study—was detected. Similarly, a number of authors have reported in the crabs *Carnicus maenas* and *Eriocheir sinensis* a decrease in food consumption when MPs were added to their food [48] and water [49]. The species studied and the means of administration (water vs. food), as well as the concentrations of MPs in water (40 µg L^−1^ in the study by Yu et al., [49]), could explain these differences.

Recent studies of fish have highlighted the complex relationship that exists between MPs and Cd [50,51], and some authors even indicated that this interaction may in some way be beneficial for aquatic organisms [52]. Thus, this complexity requires further study if clear conclusions are to be drawn [50,51,52]. In our study, the presence of MPs in water did not affect the accumulation of Cd in the haemolymph or in any of the studied tissues as no statistically significant differences in Cd concentrations between the groups that consumed tuna liver were found. However, a number of contrasting results have been published. Wen et al. [50], for example, indicated that an increase in the concentrations of MPs led to a decrease in the Cd accumulation in tissues of *Symphysodon aequifasciatus* since there was a reduction in the amount of metallothioneins, proteins that retain certain toxic elements including Cd. However, Lu et al. [53] reported the opposite in zebrafish (*Danio rerio*), in which there was an increase in Cd accumulation. The species (fish), the concentrations of MPs and Cd, and how the metal was administrated could all have led to these differences. However, in a recent study, Munuera et al. [25] found no differences in the accumulation of another trace toxic element (Pb) in blue crabs exposed to the same concentrations of MPs (25 µg L^−1^). Thus, differences in the responses to the MPs could be associated with the species.

### 4.2. Distribution, Accumulation, and Excretion of Cd

The hepatopancreas had the highest concentrations of Cd, followed by the gills (Table 2). In marine organisms, the digestive gland has been identified as the main storage and detoxification organ for metals, and more toxicants accumulate here than in gills [34,42,54,55]. Both the hepatopancreas and gills are directly involved in the uptake, storage, and excretion of metals due to their role in metallothionein synthesis [56]. Martins et al. [57] reported that there were higher concentrations of metallothioneins in hepatopancreas than in gills, which could explain the higher Cd concentrations detected in the former tissue.

Muscle concentrations of Cd were lower than those found in the hepatopancreas and gills but higher than those present in haemolymph in two of the three groups of crabs that consumed tuna liver (Table 2). The different Cd concentrations in the haemolymph and tissues could be due to the different routes of Cd exposure. Thus, in the study by Wiech et al. [58], the haemolymph of crabs that were given Cd in their diets also had the lowest Cd concentrations. According to Warner [59], the metal present in ingested food is absorbed directly from the midgut into the hepatopancreas, whereas the Cd present in the water is absorbed mainly through the gills and first enters the haemolymph before being redistributed to the digestive tract [60]—and may even be present in somewhat higher concentrations in the haemolymph than in muscles [58]. However, the greater concentrations we detected in muscles than in the haemolymph coincides with the results of other studies [42,61].

The highest Cd concentrations in faeces were found in the group exposed to virgin polyethylene (MPP 1241), although no statistically significant differences between the three experimental groups were found. Cadmium concentrations in faeces were extremely high in crabs from the experiment, which would rule out a generalised distribution and could explain the lack of correlation between biometric data and Cd concentrations in haemolymph and tissues, as previously reported by other authors [25]. Rainbow [62] stated that crustaceans can assimilate non-essential metals without excreting them (they are stored bound to metallothioneins) or can excrete them if there is an excess in a compartment. In this case, no changes occur in tissue concentrations since the rates of metal absorption and excretion tend to balance out. As indicated above, the concentrations of metallothioneins are higher in the hepatopancreas than in the gills, and the former tissue may act as a temporary reservoir for toxic elements such as Pb [25]. This could also occur in the case of Cd, since this metal has been described as an important inducer of metallothioneins [57,63].

### 4.3. Food Safety

The Cd concentrations found in the muscles of the crabs from the three groups that consumed tuna liver were not of food safety concern as they did not exceed the limits established by the European Regulation (EU) 2021/1323 [13], which states that Cd in the meat of the appendices and abdomen of crustaceans must not exceed 0.5 µg g^−1^. However, it is advisable to remove soft tissues such as the hepatopancreas since, as indicated by the European Commission’s Directorate-General for Health and Consumer Protection (DGSANCO) [16], these tissues can store high concentrations of metals, a fact verified in the crabs in our experiment that consumed tuna liver (Table 2).

The Cd concentrations in the hepatopancreas and gills of the control group were significantly and positively correlated (r = 0.782, *p* < 0.05). This result coincides with other studies [33,64] and is explained by the ‘open’ circulation system in crabs, which means that once in the haemolymph, Cd is distributed throughout the crab’s internal tissues [65]. However, the most remarkable finding was observed in specimens exposed to oxidised MPs, where a positive correlation between hepatopancreas and muscles was found. In the marine environment, plastics break down by photo-oxidation [66], and MP particles account for a significant fraction in the oceans [67]. *A priori*, this could imply an increased public health risk since if oxidised MPs are present, Cd can be transported to another edible part of the body (muscle) for which no health recommendations have been made. However, as we were able to verify in our study, the concentrations attained in muscle tissue (averages from 0.031 to 0.037 µg g^−1^, with a maximum of 0.083 µg g^−1^) were far below the limit considered in the current legislation (0.5 µg g^−1^), although the connection between the hepatopancreas and muscles should be taken into account in future studies.

## 5. Conclusions

In conclusion, the blue crab has proven to be a suitable organism for studies of food safety and environmental contaminants as they can be kept for long periods under controlled laboratory conditions. As scavengers, blue crabs can consume the viscera of large fish species, which can lead to high accumulations of Cd in internal tissues such as the hepatopancreas that exceed the maximum limit established by the current legislation for this metal. The presence of both virgin and oxidised MPs did not influence Cd accumulation in crab haemolymph and tissues, and therefore is not a risk factor for the accumulation of this pollutant. In addition, the state of the MP (virgin vs. oxidised) caused non-statistical differences in the response of crabs in terms of food consumption and tissue Cd concentrations; nevertheless, the observed trend should be checked in future long-term studies. Finally, although the Cd accumulation in muscles did not exceed the maximum limit permitted by legislation, the relationship between this tissue and the hepatopancreas in the presence of oxidised MPs should be the subject of future studies.

## Figures and Tables

**Table 1:** Descriptive statistics of biometric measurements of blue crab from the four groups.

	Weight	Length	Width
Control	32.959 ± 12.416	3.963 ± 0.490	7.838 ± 1.194
AQUATEX^®^ 230	30.202 ± 8.930	3.963 ± 0.403	7.425 ± 0.924
MPP 1241	33.648 ± 16.506	3.988 ± 0.696	7.550 ± 1.606
Zero-hour	25.664 ± 7.407	3.563 ± 0.385	7.038 ± 0.852
Whole population	30.618 ± 11.700	3.838 ± 0.507	7.463 ± 1.160

Data: mean ± standard deviation; weight = g; and length and width = cm.

**Table 2:** Cadmium concentration (μg g^−1^) in haemolymph (ww), tissues (ww), and faeces (dw) of blue crabs.

	Haemolymph	Hepatopancreas	Gills	Muscles	Faeces
Control	0.011 ± 0.010 ^A,B,(C)^ (nd-0.031)	10.424 ± 3.137 ^A,D,E^ (3.639–13.694)	0.503 ± 0.137 ^B,D,F^ (0.223–0.647)	0.032 ± 0.020 ^(C),E,F^ (0.010–0.076)	8.250 ± 0.014 (8.240–8.259)
Aquatex	0.095 ± 0.227 ^A,B^ (0.002–0.654)	13.666 ± 4.729 ^A,D,E^ (7.025–21.208)	0.539 ± 0.156 ^B,D,F^ (0.320–0.871)	0.031 ± 0.012 ^E,F^ (0.019–0.058)	8.953 ± 1.339 (8.123–10.498)
MPP	0.014 ± 0.010 ^A,B,(C)^ (0.002–0.029)	10.950 ± 2.771 ^A,D,E^ (5.010–14.123)	0.501 ± 0.129 ^B,D,F^ (0.285–0.681)	0.037 ± 0.021 ^(C),E,F^ (0.020–0.083)	12.181 ± 5.776 (5.821–17.102)
Zero-hour	nd	0.121 ± 0.080 (0.037–0.293)	nd	nd	nd

For the same group, the statistical differences in Cd concentrations between tissues are shown with capital letters in superscript; letter in brackets indicates marginally significant differences (*p* = 0.05–0.100); and nd = not detected.

## Data Availability

Data sharing not applicable.
